# State sensitivity and five-year longitudinal stability of resting-state EEG biomarker candidates in healthy adults

**DOI:** 10.3389/fnagi.2026.1885392

**Published:** 2026-06-18

**Authors:** Ye-Ji Park, Beom-Jin Kwon, Hoo-Joon Lee, Jin-Hyoung Jeong

**Affiliations:** 1 Department of Biomedical Engineering, Catholic Kwandong University, Gangneung-si, Gangwon-do, Republic of Korea; 2 Department of Health Care Management, Catholic Kwandong University, Gangneung-si, Gangwon-do, Republic of Korea

**Keywords:** alpha peak frequency, alpha power, aperiodic exponent, biomarker, EEG, resting-state EEG, state sensitivity, test–retest reliability

## Abstract

Resting-state EEG is widely used to investigate individual differences in brain function, as it allows repeated measurements with high temporal resolution and minimal task demands. However, validating EEG metrics as biomarker candidates requires more than demonstrating cross-sectional differences at a single time point. It is necessary to evaluate whether each metric is sensitive to transient state changes, such as eyes-open (EO) and eyes-closed (EC) conditions, and whether individual differences remain stable across long-term follow-up. This study investigated EO/EC state sensitivity and five-year longitudinal stability of posterior alpha relative power, alpha peak frequency, theta/beta ratio, and aperiodic exponent using publicly available resting-state EEG data from healthy adults. State sensitivity was evaluated using within-participant EO/EC differences and Cohen’s *d*, while longitudinal stability was assessed using intraclass correlation coefficients (ICC), baseline-follow-up scatter plots, and Bland–Altman plots. The analysis also examined whether the findings were preserved across reduced-channel configurations. Posterior alpha relative power showed the strongest EO/EC state sensitivity (Cohen’s *d* = 1.553) and high five-year longitudinal stability (ICC = 0.843). Alpha peak frequency showed small state sensitivity (Cohen’s *d* = 0.238) and moderate longitudinal stability (ICC = 0.734). Theta/beta ratio increased under the EO condition and showed small-to-moderate state sensitivity (Cohen’s *d* = −0.342) and good longitudinal stability (ICC = 0.772), although outlier sensitivity analysis indicated that its individual-level stability should be interpreted cautiously. Aperiodic exponent also increased under the EO condition and showed moderate-to-large state sensitivity (Cohen’s *d* = −0.761), but its five-year stability was moderate (ICC = 0.668). These findings indicate that resting-state EEG biomarkers should be evaluated separately for state sensitivity, longitudinal stability, and robustness under reduced-channel conditions. Posterior alpha relative power emerged as the most robust candidate marker in healthy adults, but it should be interpreted as a candidate EEG marker rather than as a validated clinical biomarker.

## 1 Introduction

Resting-state EEG is widely utilized across various domains—including studies on healthy aging, psychiatric and neurological disorders, and long-term follow-ups—due to its task-free nature and suitability for repeated measurements. The resting-state EEG spectrum exhibits highly stable signal properties sufficient for individual identification. Furthermore, spectral power and alpha-band metrics have demonstrated robust intra-individual stability across repeated assessments (Näpflin et al., 2007; Popov et al., 2023). However, cross-sectional group or condition differences alone are insufficient to establish EEG metrics as reliable biomarkers. To qualify as valid biomarker candidates, these metrics must be comprehensively evaluated regarding their sensitivity to transient state changes, test–retest reliability, and longitudinal stability (McKeown et al., 2024; Politanskaia et al., 2026; Henao Isaza et al., 2026; Martin et al., 2025).

As one of the most prominent rhythms in resting-state EEG, alpha activity exhibits well-established reactivity; specifically, occipital alpha power significantly increases during eyes-closed (EC) conditions and attenuates during eyes-open (EO) conditions (Bazanova and Vernon, 2014; Barry et al., 2007). Consequently, posterior alpha relative power serves as a highly sensitive metric for state changes, particularly those modulated by visual input. Conversely, the individual alpha frequency (IAF) or alpha peak frequency (APF) has been proposed as a relatively stable trait marker. These metrics are strongly associated with cognitive performance, aging, and inter-individual neurophysiological profiles (Grandy et al., 2013; Haegens et al., 2014; Chiang et al., 2011; Klimesch, 1999; Surwillo, 1961). Nevertheless, because alpha-band metrics can still fluctuate based on the subject’s state, task context, and the specific frequency boundaries analyzed, it is imperative to evaluate alpha power and peak frequency independently (Haegens et al., 2014; Chiang et al., 2011).

The theta/beta ratio (TBR) is a widely utilized EEG metric in studies investigating attentional control, arousal levels, and ADHD. However, because it integrates distinct broad frequency bands into a single composite index, its values are highly susceptible to variations in age, cognitive state, preprocessing methods, electrode montages, and analytical pipelines (Arns et al., 2013; Snyder and Hall, 2006; Loo and Makeig, 2012; Lenartowicz and Loo, 2014; Angelidis et al., 2016; Strzelczyk et al., 2026; Gholamali Nezhad et al., 2025). Recent studies tend to interpret TBR as an auxiliary indicator reflecting specific subgroups or state characteristics rather than as a single diagnostic biomarker. In addition to attention-related research, TBR has also been examined in the context of cognitive aging, mild cognitive impairment, and dementia-related processes (Martin et al., 2023). Elevated TBR has been reported in cognitively impaired groups, suggesting that this ratio may reflect broader alterations in cortical slowing or cognitive vulnerability rather than only attentional dysregulation. Therefore, TBR should be evaluated for long-term stability, intra-individual variability, and outlier sensitivity along with statistical significance.

In recent EEG analysis, there is increasing interest in interpreting the power spectrum by dividing it into a periodic component and an aperiodic component in addition to the traditional band power (Donoghue et al., 2020; Gerster et al., 2022). Aperiodic activity or 1/f-like activity has been associated with age, cognitive function, and overall levels of neural activity, and has been discussed as an indicator related to excitation-inhibition balance or neural noise (Bai et al., 2024; Karalunas et al., 2022; Miller et al., 2009; Voytek et al., 2015; Dave et al., 2018; He, 2014). However, because the aperiodic exponent can be affected by frequency range, artifact processing, baseline correction, and spectral parameterization methods, reliability and stability reviews are required before using it as a trait-like biomarker for long-term tracking (McKeown et al., 2024; Gerster et al., 2022; Gyurkovics et al., 2021).

Considering practical applicability, it is also important to determine whether the findings remain consistent across reduced-channel configurations when the number of channels is reduced. High-density EEG provides abundant spatial information, but in clinical settings, community-based measurements, remote monitoring, and wearable EEG environments, reduced-channel EEG may be more practical because of lower cost, ease of application, and setup time (Casson, 2019; Krigolson et al., 2017; Mihajlović et al., 2015). In addition, EEG channel selection and channel reduction have been consistently addressed in BCI, clinical classification, and wearable EEG studies (Li et al., 2026; Zuo et al., 2024; Maheshwari et al., 2025; Khanam et al., 2025; Özkahraman et al., 2025; Alotaiby et al., 2015; Lotte et al., 2018). Therefore, ensuring that the results obtained using 64 channels are also maintained at 32, 19, 8, and 4 channels is a necessary step in evaluating the practicality of EEG biomarkers.

Using publicly available resting-state EEG data from healthy adults, this study compared the eyes-open/eyes-closed state sensitivity and long-term stability of the posterior alpha relative power, alpha peak frequency, theta/beta ratio, and aperiodic exponent over approximately 5 years using a consistent framework. In addition, it was confirmed that the 64-channel analysis results were maintained across reduced-channel configurations. The purpose of this study is not to simply classify each EEG metric as superior or inferior, but to distinguish between metrics suitable for tracking state changes and those suitable for long-term individual difference evaluation.

## 2 Research method

### 2.1 Dataset and participants

This study was a secondary analysis of a publicly available resting-state EEG dataset (OpenNeuro ds005385). The dataset contains resting-state EEG recordings from healthy adults across the adult lifespan, including eyes-open and eyes-closed conditions at baseline and approximately five-year follow-up. The analytic sample included 608 participants at baseline and 208 participants at follow-up. Baseline participants were 20–70 years old, with a mean age of 44.07 ± 14.51 years. The sample included 376 female and 232 male participants.

For the present analysis, eyes-open and eyes-closed resting-state EEG recordings were used to evaluate state sensitivity, while participants with both baseline and follow-up recordings were used to evaluate five-year longitudinal stability. When repeated pre/post acquisition files were available within the same session and condition, EEG features were averaged within each subject-session-condition-channel subset before statistical analysis to avoid inflating the sample size.

### 2.2 EEG preprocessing

Raw EEG files were processed using MNE-Python. EEG signals were loaded from the public dataset, restricted to EEG channels, band-pass filtered from 1 to 40 Hz, and re-referenced to the average reference. Power spectral density was estimated using Welch’s method within the 1–40 Hz frequency range. The present study did not involve new EEG acquisition or manual clinical assessment; all analyses were performed on de-identified publicly available EEG data.

For each file, spectral features were extracted separately for the original 64-channel montage and reduced-channel configurations. The primary high-density analysis used all available EEG channels. Reduced-channel analyses were conducted using 32-channel, 19-channel, 8-channel, and 4-channel configurations to evaluate whether the main findings were preserved in low-channel settings.

### 2.3 Spectral feature extraction

Four resting-state EEG biomarker candidates were extracted: posterior alpha relative power, alpha peak frequency, theta/beta ratio, and aperiodic exponent. Posterior alpha relative power was calculated from posterior channels, including parietal, parieto-occipital, and occipital electrodes. Alpha relative power was defined as alpha-band power divided by total spectral power in the analyzed 1–40 Hz range. Alpha peak frequency was defined as the frequency showing the maximum spectral power within the alpha band. Theta/beta ratio was calculated as theta-band power divided by beta-band power.

The aperiodic exponent was estimated using FOOOF/specparam-style spectral parameterization of the power spectrum. Spectral parameterization was performed within the 2–40 Hz range, with peak width limits of 1.0–8.0 Hz, a maximum of six peaks, a minimum peak height of 0.1, and a fixed aperiodic mode. The aperiodic exponent was included because it captures non-oscillatory spectral structure that may provide information complementary to conventional band-power measures.

Alpha peak amplitude and aperiodic offset were not included as primary outcomes because the present study aimed to compare a focused set of conceptually distinct and commonly interpretable EEG measures. Alpha peak amplitude partly overlaps with alpha power, whereas aperiodic offset reflects broadband power level and can be sensitive to reference choice, noise floor, and preprocessing. The omission of these additional spectral variables is acknowledged as a limitation.

### 2.4 State sensitivity analysis

State sensitivity was evaluated by comparing eyes-closed and eyes-open conditions at baseline. For each EEG measure, paired comparisons were conducted within participants. Cohen’s *d* was calculated as the standardized within-participant difference between eyes-closed and eyes-open conditions. Positive values indicate higher values in the eyes-closed condition, whereas negative values indicate higher values in the eyes-open condition. Statistical significance was evaluated using paired tests, and the magnitude of the effect was interpreted alongside the *p*-value.

### 2.5 Longitudinal stability analysis

Five-year longitudinal stability was evaluated in participants with both baseline and follow-up recordings. The primary reliability metric was the intraclass correlation coefficient. ICC was calculated using a two-way mixed-effects, absolute-agreement, single-measure model [ICC (A, 1)]. ICC values were interpreted using commonly applied reliability criteria: values below 0.50 were considered poor, values between 0.50 and 0.75 moderate, values between 0.75 and 0.90 good, and values above 0.90 excellent. Baseline-follow-up scatter plots and Bland–Altman plots were also examined to assess longitudinal agreement and individual-level variability.

### 2.6 Outlier sensitivity analysis

Because theta/beta ratio can be sensitive to extreme observations, an additional outlier sensitivity analysis was performed. Potential theta/beta ratio outliers were identified using the 1.5 × interquartile range criterion based on baseline eyes-closed values. State sensitivity and longitudinal stability analyses were repeated after excluding these participants. This analysis was used to determine whether the theta/beta ratio findings were driven by a small number of extreme observations.

### 2.7 Age-adjusted sensitivity analysis

Because age can influence alpha power, alpha peak frequency, theta/beta ratio, and aperiodic spectral characteristics, age-adjusted sensitivity analyses were performed. Age was included as a covariate in additional models evaluating EO/EC condition effects. These analyses were used to determine whether the main state-sensitivity findings were preserved after accounting for age-related variation.

### 2.8 Reduced-channel analysis

The same feature extraction and statistical analyses were repeated for reduced-channel configurations. The retained channel sets were summarized in a channel montage table. The 64-channel analysis used all available EEG channels. The 32-channel configuration retained 30 available electrodes in the present dataset, while the 19-channel, 8-channel, and 4-channel configurations retained standard reduced montages centered on frontal, central, parietal, and occipital coverage. For consistency with the predefined analysis plan, this configuration is referred to as the 32-channel subset throughout the manuscript, although 30 electrodes were available in the present dataset. For each channel configuration, state sensitivity was quantified using Cohen’s *d* and five-year longitudinal stability was quantified using ICC (A, 1). The retained electrodes for each reduced-channel configuration are provided in Supplementary Table 1.

### 2.9 Biomarker characterization map

A biomarker characterization map was generated to visualize each EEG measure according to two dimensions: EO/EC state sensitivity and five-year longitudinal stability. The absolute magnitude of Cohen’s *d* was plotted on the x-axis, and ICC (A, 1) was plotted on the y-axis. Reference lines were used as heuristic visual aids rather than strict diagnostic thresholds. Therefore, the interpretation emphasized the continuous values of Cohen’s *d* and ICC rather than categorical quadrant assignment alone.

## 3 Results

### 3.1 State sensitivity under eyes-open/closed condition

**Figure 1:**
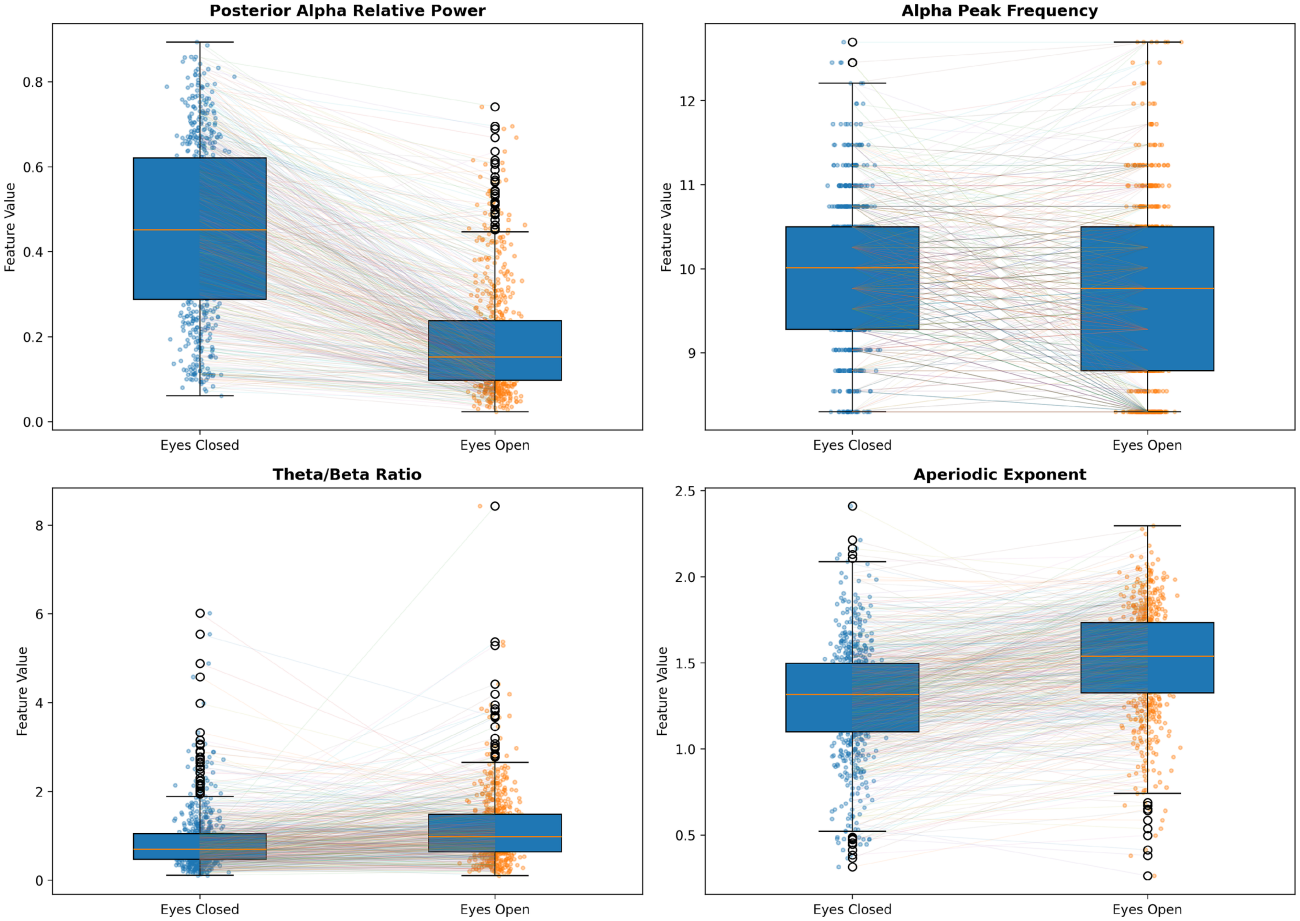
Paired distributions of EEG biomarker candidates between eyes-closed and eyes-open conditions at baseline. Lines indicate within-participant EO/EC changes. Positive Cohen’s *d* values indicate higher values in eyes-closed conditions, whereas negative values indicate higher values in eyes-open conditions.

All four EEG measures showed significant EO/EC condition differences, but the magnitude and direction of the effects differed across measures (Table 1, Figure 1). Posterior alpha relative power showed the strongest state sensitivity, with higher values in the eyes-closed condition than in the eyes-open condition (EC mean = 0.453, EO mean = 0.190, Cohen’s *d* = 1.553, *p* < 0.001). This indicates a very large alpha reactivity effect.

**Table 1:** State sensitivity of resting-state EEG biomarker candidates between eyes-closed and eyes-open conditions.

Biomarker candidate	EC mean	EO mean	Cohen’s *d*	Interpretation
Posterior alpha relative power	0.453	0.190	1.553	Very large EC > EO effect
Aperiodic exponent	1.288	1.511	−0.761	Moderate-to-large EO > EC effect
Theta/beta ratio	0.862	1.157	−0.342	Small-to-moderate EO > EC effect
Alpha peak frequency	9.946	9.702	0.238	Small EC > EO effect

Aperiodic exponent also showed a marked EO/EC difference, but in the opposite direction, with higher values in the eyes-open condition than in the eyes-closed condition (EC mean = 1.288, EO mean = 1.511, Cohen’s *d* = −0.761, *p* < 0.001). Thus, the aperiodic exponent was not independent of resting-state condition and should be interpreted as a state-sensitive spectral measure.

Theta/beta ratio was also higher in the eyes-open condition than in the eyes-closed condition (EC mean = 0.862, EO mean = 1.157, Cohen’s *d* = −0.342, *p* < 0.001), indicating a small-to-moderate state effect. Alpha peak frequency showed a statistically significant but small EO/EC effect (EC mean = 9.946 Hz, EO mean = 9.702 Hz, Cohen’s *d* = 0.238, *p* < 0.001). Overall, posterior alpha relative power showed the clearest and largest EO/EC state sensitivity.

### 3.2 Distribution and intra-individual variation across conditions

The paired distribution plots supported the numerical results (Figure 1). Posterior alpha relative power decreased consistently across most participants when switching from eyes-closed to eyes-open, which aligns with the very large effect size reported in Table 1. Alpha peak frequency showed a smaller EO/EC shift. In contrast, theta/beta ratio and aperiodic exponent tended to increase under eyes-open conditions, indicating that these indices are influenced by resting-state condition and should not be interpreted as purely trait-like measures.

### 3.3 Five-year long-term stability

**Figure 2:**
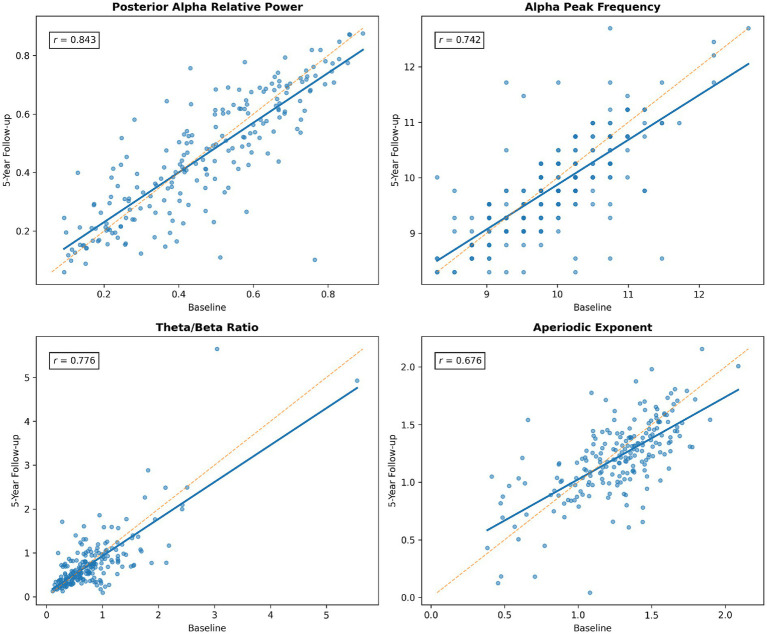
Baseline–follow-up scatter plots of EEG biomarker candidates. Each point represents one participant with data at both baseline and approximately five-year follow-up. The dashed diagonal line indicates equality between the two time points.

Five-year longitudinal stability differed across the four EEG measures (Table 2, Figure 2). Posterior alpha relative power showed the highest stability among the four measures (ICC = 0.843, *r* = 0.843), indicating that individual differences in this measure were substantially preserved across the five-year interval. Theta/beta ratio also showed good longitudinal stability (ICC = 0.772, *r* = 0.776). Alpha peak frequency showed moderate stability (ICC = 0.734, *r* = 0.742). Aperiodic exponent showed moderate but lower stability than posterior alpha relative power and theta/beta ratio (ICC = 0.668, *r* = 0.676). These findings indicate that posterior alpha relative power was the most stable measure over the five-year follow-up, whereas aperiodic exponent was more state-sensitive than trait-stable in this dataset.

**Table 2:** Five-year longitudinal stability of resting-state EEG biomarker candidates.

Biomarker candidate	ICC (A, 1)	Pearson’s *r*	Reliability grade
Posterior alpha relative power	0.843	0.843	Good
Theta/beta ratio	0.772	0.776	Good
Alpha peak frequency	0.734	0.742	Moderate
Aperiodic exponent	0.668	0.676	Moderate

### 3.4 Baseline-follow-up relationships

As shown in Figure 2, all four EEG measures showed positive baseline-follow-up associations. The strongest association was observed for posterior alpha relative power, followed by theta/beta ratio, alpha peak frequency, and aperiodic exponent. These scatter plots indicate that group-level longitudinal stability was present, but the degree of individual-level dispersion differed by measure.

### 3.5 Bland–Altman analysis

**Figure 3:**
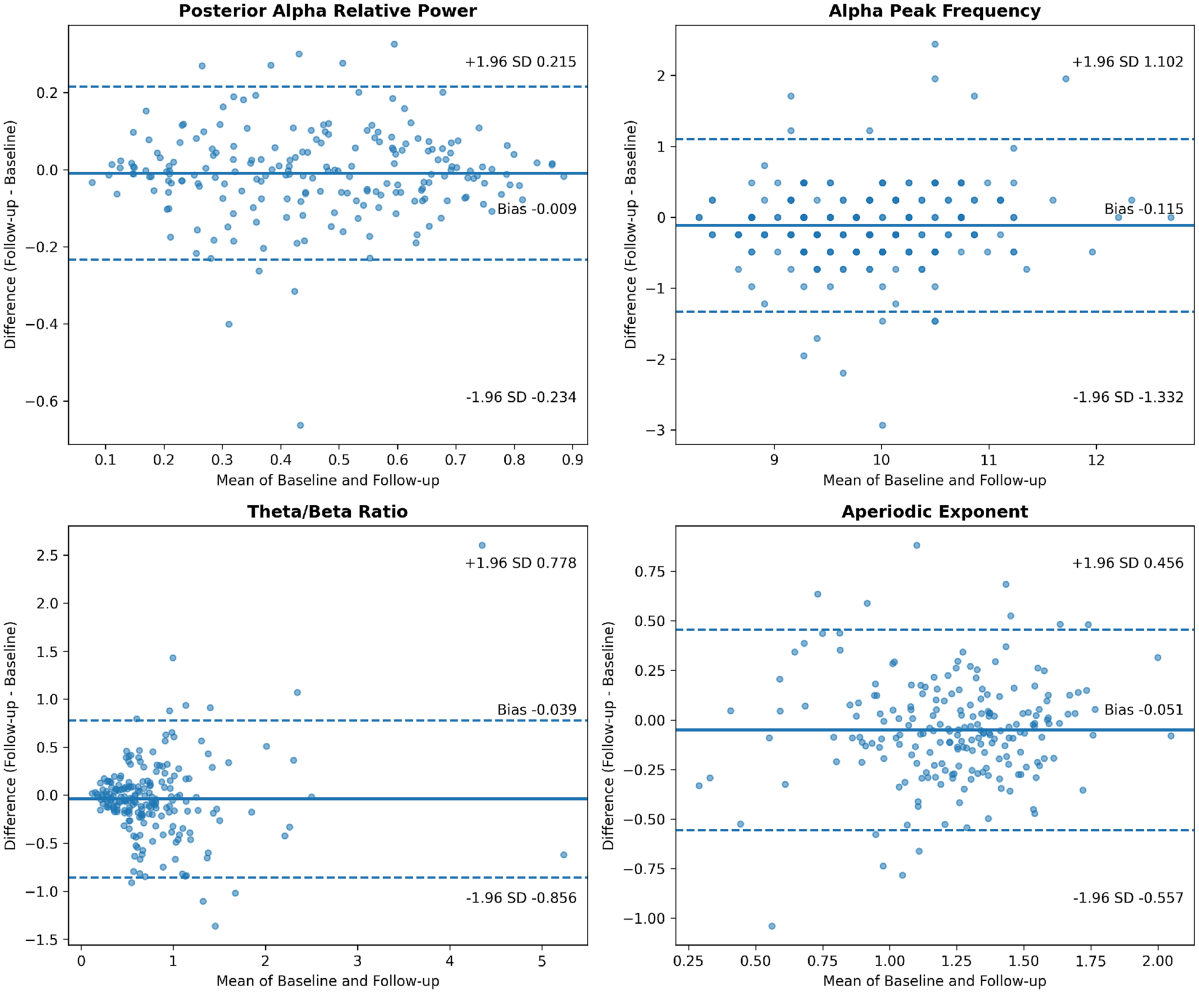
Bland–Altman plots for five-year agreement of EEG biomarker candidates. The solid line indicates mean bias, and dashed lines indicate limits of agreement.

Figure 3 shows the Bland–Altman plots for the four EEG measures. Posterior alpha relative power showed relatively stable agreement, whereas theta/beta ratio and aperiodic exponent showed wider individual-level variability. These plots supported the need to interpret ICC values together with visual agreement patterns rather than relying on reliability coefficients alone.

### 3.6 Theta/beta ratio outlier sensitivity analysis

Because theta/beta ratio can be sensitive to extreme observations, we performed an additional outlier sensitivity analysis using the 1.5 × IQR criterion. Thirty-six participants were identified as potential theta/beta ratio outliers based on baseline eyes-closed values. In the full sample, theta/beta ratio showed a small-to-moderate EO/EC effect (Cohen’s *d* = −0.342) and good five-year stability (ICC = 0.772). After excluding outliers, the EO/EC effect remained significant and became larger in magnitude (Cohen’s *d* = −0.491), whereas longitudinal stability decreased to a moderate level (ICC = 0.585). These results indicate that the theta/beta ratio findings were not solely driven by extreme observations, but the stability of this measure is sensitive to outlier handling. Therefore, theta/beta ratio should be interpreted cautiously when used for individual-level longitudinal monitoring.

### 3.7 Age-adjusted sensitivity analysis

Because age was significantly associated with the EEG measures, we performed age-adjusted sensitivity analyses. After including age as a covariate, the EO/EC condition effect remained significant for posterior alpha relative power (condition coefficient = 0.263, *p* < 0.001), alpha peak frequency (condition coefficient = 0.243, *p* < 0.001), theta/beta ratio (condition coefficient = −0.295, *p* < 0.001), and aperiodic exponent (condition coefficient = −0.223, *p* < 0.001). Age itself was also significantly associated with each measure. These results indicate that the observed EO/EC state effects were not explained solely by age-related variation.

### 3.8 Two-dimensional classification of state sensitivity and long-term stability

**Figure 4:**
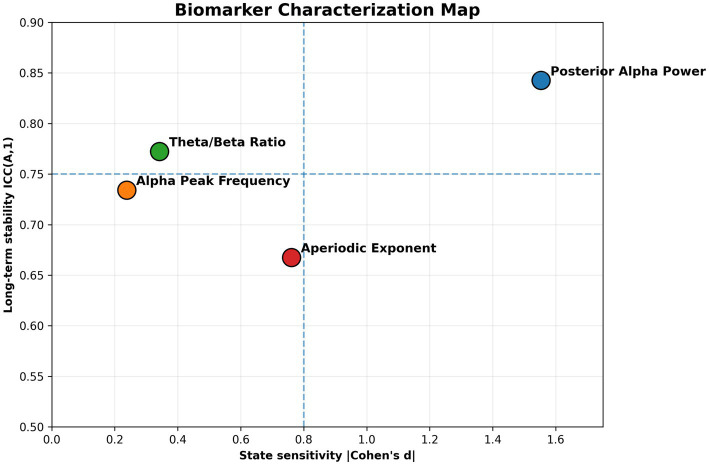
Biomarker characterization map based on EO/EC state sensitivity and five-year longitudinal stability. The *x*-axis represents the absolute magnitude of Cohen’s *d*, and the *y*-axis represents ICC (A, 1). Reference lines are visual guides rather than strict diagnostic thresholds.

The biomarker characterization map summarized the relationship between state-sensitivity magnitude and five-year longitudinal stability (Figure 4). Posterior alpha relative power was positioned as the most robust candidate measure because it combined very large state sensitivity with good longitudinal stability. Theta/beta ratio showed good full-sample stability but only small-to-moderate state sensitivity, and its interpretation was affected by outlier sensitivity. Alpha peak frequency showed small state sensitivity and moderate stability. Aperiodic exponent showed moderate-to-large state sensitivity but only moderate longitudinal stability. Therefore, the map should be interpreted as a visual summary of continuous effect-size and ICC values rather than as a strict diagnostic classification.

### 3.9 Channel-subset robustness analysis

**Figure 5:**
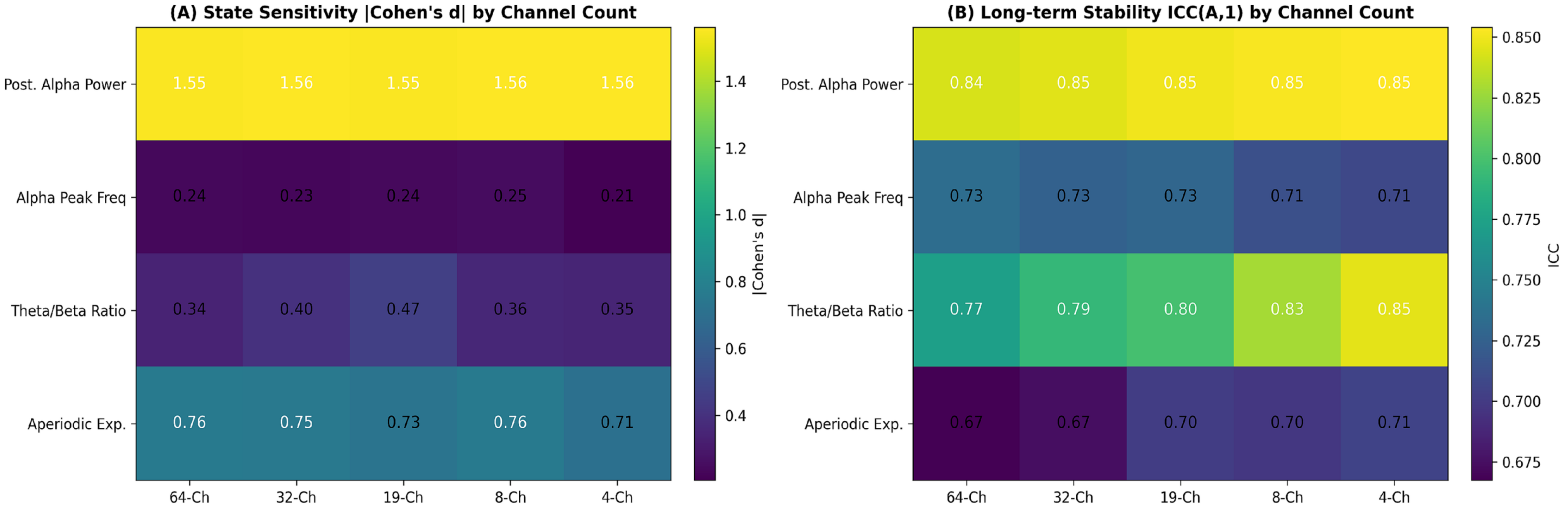
Channel subset robustness of state sensitivity and long-term stability. **(A)** Heatmap showing EO/EC state sensitivity quantified by Cohen’s d across the original 64-channel montage and reduced-channel configurations. **(B)** Heatmap showing five-year longitudinal stability quantified by ICC (A, 1) across the original 64-channel montage and reduced-channel configurations.

The channel-subset analysis showed that the overall pattern of results was preserved across the original 64-channel montage and the reduced-channel configurations (Figure 5). The retained electrodes for the original 64-channel montage and reduced-channel configurations are summarized in Supplementary Table 1. Posterior alpha relative power showed very large state sensitivity across all configurations, with Cohen’s d ranging from 1.550 to 1.560, and good longitudinal stability, with ICC values ranging from 0.843 to 0.854. This indicates that posterior alpha relative power was robust even when the number of channels was reduced. Alpha peak frequency showed consistently small state sensitivity across channel configurations (Cohen’s *d* = 0.207–0.252) and moderate longitudinal stability (ICC = 0.708–0.734). Theta/beta ratio showed small-to-moderate state sensitivity, with larger effects in the 19-channel configuration, and its ICC ranged from 0.772 to 0.847 across channel subsets. Aperiodic exponent showed moderate-to-large EO/EC sensitivity across all channel subsets (Cohen’s *d* = −0.551 to −0.761), while its ICC remained moderate (0.668–0.705). These results suggest that posterior alpha relative power was the most consistently robust measure across reduced-channel settings, whereas theta/beta ratio and aperiodic exponent were more sensitive to analytic and montage-related variation (see Table 3).

**Table 3:** Integrated classification of EEG biomarker candidates.

Biomarker	State sensitivity	Long-term stability	Overall interpretation
Posterior alpha relative power	Very large	Good	Most robust candidate dual-purpose EEG marker in healthy adults
Alpha peak frequency	Small	Good	Relatively trait-like measure with weak state response
Theta/beta ratio	Small-to-moderate	Good, but outlier-sensitive	Group-level stability with individual-level caution
Aperiodic exponent	Moderate-to-large	Moderate	State-sensitive aperiodic measure with lower long-term stability than posterior alpha power

## 4 Discussion

This study compared the state sensitivity and long-term stability of four EEG biomarker candidates within the same analysis framework using a publicly available resting-state EEG dataset of healthy adults. The revised analysis confirmed that each measure showed a distinct profile. Posterior alpha relative power showed the strongest EO/EC state sensitivity and the highest five-year stability. Alpha peak frequency showed weak state sensitivity and moderate longitudinal stability. Theta/beta ratio showed small-to-moderate state sensitivity and good full-sample stability, but its stability was sensitive to outlier handling. Aperiodic exponent showed moderate-to-large state sensitivity but only moderate five-year stability.

These findings are broadly consistent with prior resting-state EEG literature showing that alpha activity contains both state-dependent and individually stable components. The strong EO/EC effect of posterior alpha relative power agrees with the well-established alpha blocking response during eye opening, whereas its high five-year stability supports earlier reports that resting EEG spectra can contain stable individual signatures. The moderate stability of alpha peak frequency also aligns with studies describing alpha peak frequency as a trait-like neurophysiological measure, although the present results suggest that its short-term EO/EC sensitivity is limited compared with posterior alpha power. The theta/beta ratio findings should be interpreted in a broader context than attention-related research alone. Prior studies in cognitive impairment and dementia-related conditions suggest that elevated theta/beta ratio may reflect cortical slowing or cognitive vulnerability; however, the present outlier sensitivity analysis indicates that this measure may be more reliable at the group level than at the individual level. Finally, the state sensitivity and moderate longitudinal stability of the aperiodic exponent are consistent with spectral parameterization studies emphasizing that aperiodic estimates can be influenced by frequency range, preprocessing, and model settings.

The posterior alpha relative power was the most robust candidate measure in the present dataset. It showed the largest EO/EC state sensitivity and the highest five-year longitudinal stability among the four measures. This combination suggests that posterior alpha relative power can capture both short-term changes in visual/resting state and relatively stable individual differences. However, because the present study was conducted in healthy adults and did not evaluate cognitive decline, disease progression, or clinical outcomes, this measure should be interpreted as a candidate dual-purpose EEG marker rather than as a validated clinical biomarker.

The alpha peak frequency showed weak state sensitivity and moderate longitudinal stability. Therefore, this metric may be more suitable for identifying relatively stable individual neurophysiological characteristics or evaluating individual differences over a long-term follow-up than for monitoring short-term EO/EC state changes.

The theta/beta ratio showed small-to-moderate state sensitivity and good longitudinal stability in the full sample. However, the outlier sensitivity analysis changed the interpretation of this measure. After excluding participants with extreme theta/beta ratio values, the EO/EC effect remained significant, but the ICC decreased from 0.772 to 0.585. This indicates that theta/beta ratio can appear relatively stable in the full sample, but its stability estimate is sensitive to extreme observations. Therefore, theta/beta ratio may be useful for group-level characterization, but it should be applied cautiously as an individual-level longitudinal biomarker.

The aperiodic exponent showed moderate-to-large EO/EC state sensitivity but only moderate five-year longitudinal stability. This suggests that the aperiodic exponent contains meaningful state-related information in resting-state EEG, particularly in relation to EO/EC condition differences. At the same time, its lower ICC compared with posterior alpha relative power indicates that it may be less suitable as a stand-alone trait-like longitudinal marker. Therefore, the aperiodic exponent should be interpreted as a state-sensitive aperiodic measure whose longitudinal use requires careful attention to preprocessing, spectral parameterization settings, and replication across datasets.

The advantage of this study is that state sensitivity and long-term stability were evaluated together rather than using a single criterion. The biomarker characterization map can be used to determine which research purpose is most appropriate for each metric rather than simply dividing measures into good or bad categories. For example, posterior alpha relative power may be most appropriate when the goal is to track EO/EC changes or arousal-related state variation, whereas alpha peak frequency may be more appropriate when the goal is to assess relatively stable individual differences. Theta/beta ratio and aperiodic exponent require more cautious interpretation because they are more sensitive to outliers, preprocessing, or spectral parameterization choices.

The fact that the main results were maintained even in the reduced-channel subsets increases the practical relevance of this study. Posterior alpha relative power maintained very large state sensitivity and good five-year stability not only in the original 64-channel montage but also in reduced 32-, 19-, 8-, and 4-channel configurations. This suggests that this metric does not depend solely on high-density EEG equipment and may be considered first in wearable EEG or low-channel EEG-based monitoring studies. However, for actual clinical application, the same results should be verified across equipment types, electrode locations, noise levels, and preprocessing methods.

This study also has limitations. First, it is difficult to generalize the findings to clinical populations directly because the analysis was limited to healthy adults. Second, this study focused primarily on spectral features and did not include other EEG metrics, such as connectivity, complexity, and microstates. Third, the sample size at follow-up was smaller than that of the baseline assessment, so the confidence intervals for some long-term stability estimates may be wide. Fourth, the aperiodic exponent and theta/beta ratio can be influenced by preprocessing and spectral parameterization methods. In addition, although age-adjusted sensitivity analyses were performed, the present study did not model nonlinear age trajectories or interactions between age and eye condition in detail. Future studies should examine whether the state sensitivity and longitudinal stability of these EEG measures differ across younger, middle-aged, and older adult subgroups. Finally, alpha peak amplitude and aperiodic offset were not included as primary outcomes, and future work should evaluate whether these additional spectral parameters provide complementary information.

Nevertheless, this study is significant in that it compared the state sensitivity, five-year longitudinal stability, outlier robustness, age effects, and reduced-channel feasibility of EEG biomarker candidates using the same dataset and a consistent analysis framework.

## 5 Conclusion

This study compared EO/EC state sensitivity, five-year longitudinal stability, and reduced-channel robustness of resting-state EEG biomarker candidates in healthy adults. Posterior alpha relative power showed the most consistent profile, with very large EO/EC state sensitivity, good five-year stability, and robust performance across reduced-channel configurations. Therefore, posterior alpha relative power may be considered a leading candidate dual-purpose EEG marker in healthy adults.

Alpha peak frequency showed weak state sensitivity and moderate longitudinal stability, supporting its interpretation as a relatively trait-like measure rather than a strong state marker. Theta/beta ratio showed small-to-moderate state sensitivity and good stability in the full sample, but outlier sensitivity analysis indicated that its individual-level longitudinal interpretation requires caution. Aperiodic exponent showed moderate-to-large state sensitivity but only moderate long-term stability, suggesting that it captures state-related aperiodic information but should not be treated as a simple trait-like biomarker without further validation.

Overall, resting-state EEG biomarker candidates should not be evaluated using a single criterion. State sensitivity, longitudinal stability, outlier robustness, age effects, and reduced-channel feasibility should be considered together when selecting EEG measures for longitudinal aging research and low-channel EEG applications.

## Data Availability

Publicly available datasets were analyzed in this study. This data can be found here: The dataset analyzed in this study is publicly available in OpenNeuro under the title “Resting-state EEG data before and after cognitive activity across the adult lifespan and a 5-year follow-up.” Dataset ID: ds005385, version 1.0.3. Direct link: https://openneuro.org/datasets/ds005385/versions/1.0.3.
