# Supplementary material for: State sensitivity and five-year longitudinal stability of resting-state EEG biomarker candidates in healthy adults
Source: Front Aging Neurosci. 2026 Jun 18;18:1885392. doi: 10.3389/fnagi.2026.1885392 (PMC13323486; doi:10.3389/fnagi.2026.1885392)
Supplement: Supplementary file 1 [file Table_1.DOCX]

Supplementary Table 1. Retained channels in each EEG montage

| **Montage** | **Available  channels** | **Retained channels** | **Posterior channels used** |
| --- | --- | --- | --- |
| 64-channel | 64 | Fp1, Fp2, F7, F3, Fz, F4, F8, FC5, FC1, FC2, FC6, T7, C3, Cz, C4, T8, TP9, CP5, CP1, CP2, CP6, TP10, P7, P3, Pz, P4, P8, PO9, O1, Oz, O2, PO10, AF7, AF3, AF4, AF8, F5, F1, F2, F6, FT9, FT7, FC3, FC4, FT8, FT10, C5, C1, C2, C6, TP7, CP3, CPz, CP4, TP8, P5, P1, P2, P6, PO7, PO3, POz, PO4, PO8 | P3, Pz, P4, O1, Oz, O2, PO3, POz, PO4 |
| 32-channel intended subset | 30 available | Fp1, Fp2, F7, F3, Fz, F4, F8, FC5, FC1, FC2, FC6, T7, C3, Cz, C4, T8, CP5, CP1, CP2, CP6, P7, P3, Pz, P4, P8, O1, Oz, O2, PO3, PO4 | P3, Pz, P4, O1, Oz, O2, PO3, PO4 |
| 19-channel | 19 | Fp1, Fp2, F7, F3, Fz, F4, F8, T7, C3, Cz, C4, T8, P7, P3, Pz, P4, P8, O1, O2 | P3, Pz, P4, O1, O2 |
| 8-channel | 8 | Fz, Cz, P3, Pz, P4, O1, Oz, O2 | P3, Pz, P4, O1, Oz, O2 |
| 4-channel | 4 | P3, P4, O1, O2 | P3, P4, O1, O2 |
